# Fluorescence of Helical Molecular Springs Under High Pressure

**DOI:** 10.1002/anie.202500923

**Published:** 2025-03-18

**Authors:** Jiaxu Liang, Cheng‐Wei Ju, Zonghang Liu, Hailong Li, Aigerim Karina, Tobias Eklund, Wenhao Zheng, Katrin Amann‐Winkel, Weizhao Cai, Manfred Wagner, Zijie Qiu, Tanja Weil, Klaus Müllen

**Affiliations:** ^1^ Max Planck Institute for Polymer Research Ackermannweg 10 55128 Mainz Germany; ^2^ Pritzker School of Molecular Engineering University of Chicago Chicago Illinois 60637 USA; ^3^ School of Science and Engineering Shenzhen Institute of Aggregate Science and Technology The Chinese University of Hong Kong Shenzhen (CUHK‐Shenzhen) Guangdong 518172 P.R. China; ^4^ Department of Physics Stockholm University Roslagstullsbacken 21 Stockholm 10691 Sweden; ^5^ Institute of Physics Johannes Gutenberg University Mainz Staudingerweg 7 55128 Mainz Germany; ^6^ School of Materials and Energy University of Electronic Science and Technology of China Chengdu 611731 P.R. China; ^7^ Department of Chemistry University of Cologne Greinstr. 4–6 50939 Cologne Germany

**Keywords:** Π‐Extended helicene, Diamond anvil cell, High‐pressure fluorescence, Molecular spring

## Abstract

Although the unique structure of helicenes resembles molecular springs, the effects of their extension–contraction cycles on their properties have rarely been explored. Here, we investigated the fluorescence of two π‐extended [*n*]helicenes with different helical lengths *n*, named **[7]** and **[9]**, under high pressures in a diamond anvil cell. The experimental results showed that compound **[9]**, with a longer helical length, exhibited a more sensitive fluorescence response than **[7]** in both crystalline and solvated states upon compression. Theoretical calculations reveal that π–π overlapping at their helices in these molecular springs provides an additional contribution to their fluorescence properties under compression when the overlap becomes sufficiently strong. Our results provide insights into structure–property relationships of helical molecules under high‐pressure conditions and verify the potential of helicenes as molecular springs for future applications in molecular machines.

## Graphical abstract

A pair of π‐extended [*n*]helicenes (*n* = 7 and 9) were selected as model systems for molecular springs to study the relationship between optical properties and molecular structures at high pressure in a diamond anvil cell. Based on experimental and simulation results, the fluorescence properties of these molecular springs were found to be influenced not only by the intermolecular interaction but also by the unique intramolecular π–π interactions between their overlapping helices.

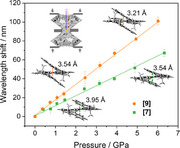

[*n*]Helicenes are nonplanar polycyclic aromatic hydrocarbons (PAHs) with *n* arylene rings fused in their *ortho* positions.^[^ ^1^ ^]^ The helically twisted structures lead to a series of exciting mechanical, electronic, and optical properties.^[^ ^2^ ^]^ Such unique helical structures have prompted scientists to envision their applications as molecular springs in molecular machines. Recently, Nakakuki et al. described a flexible π‐expanded helicene as a soft molecular spring.^[^ ^3^ ^]^ According to their simulations, the potential energies of different helicenes increase upon elongation, while the force constants of these molecular springs strongly depend on their helical diameters. There is, thus, a striking similarity to the mechanical properties of macroscopic spring materials in daily life. The elastic extension–contraction motion of these molecular springs could be triggered by external stimuli. For example, by a stepwise chemical reduction with alkali metals, double helicenes could be successively compressed, exhibiting profound bond length alterations and conformational changes.^[^ ^4^, ^5^, ^6^ ^]^ The spring‐like behavior is of great importance in helical smart materials for sensing, chemical separation, and nano‐mechanical actuators.^[^ ^7^ ^]^ Experimental studies to investigate the effect of such extension–contraction motion on their properties, however, have remained rare due to limited approaches for in situ characterization while applying force on such molecular springs.

Pressure has profound effects on chemical bonds, phase transformations, and thermodynamic properties of materials. In situ characterizations have been developed to study the structural changes of biomacromolecules, organic molecular crystals, and inorganic materials under high pressures.^[^ ^8^, ^9^, ^10^, ^11^, ^12^, ^13^ ^]^ Compared to the Raman signal, fluorescence is generally more sensitive to molecular packing and structural changes. Therefore, various fluorescent materials have been investigated under pressure, including polycyclic aromatic molecules,^[^ ^14^, ^15^ ^]^ luminogens with rotational and/or vibrational mobile groups,^[^ ^16^, ^17^ ^]^ donor–acceptor type fluorophores,^[^ ^18^, ^19^ ^]^ and covalent organic frameworks.^[^ ^20^, ^21^ ^]^ Different mechanisms have been proposed to explain their emission behavior upon compression, such as compact π–π stacking,^[^ ^22^ ^]^ formation of excimers or exciplexes,^[^ ^23^, ^24^ ^]^ enhanced intermolecular hydrogen bonds,^[^ ^25^ ^]^ planarization of twisted molecular conformation,^[^ ^26^, ^27^ ^]^ or promotion of intramolecular charge transfer effects.^[^ ^18^, ^19^ ^]^ However, high‐pressure studies for helically twisted PAHs with intramolecular structural changes remain unexplored until now. Measuring the fluorescence of helicenes under high pressure is expected to shed light on structural changes and thus experimentally explore their potential as molecular springs.

Recently, we synthesized a pair of π‐extended [*n*]helicenes (*n* = 7 and 9) with the same π‐extension motif but different helical lengths *n*.^[^ ^28^ ^]^ These π‐extended helicenes (denoted as **[7]** and **[9]**, respectively) were selected as models of helical molecular springs, and their fluorescence signals were utilized to investigate the structural changes under high pressure in a diamond anvil cell (DAC) (Figure 1). Compound **[9]** showed a larger shift in its fluorescence wavelength than **[7]** in both crystalline and solvated states. The different structure–property relations of **[7]** and **[9]** under high pressures were further elucidated by theoretical calculations using density functional theory (DFT) and hybrid quantum mechanics/molecular mechanics (QM/MM) methods. Intriguingly, the unique structures of overlapping helices were proposed to play an important role in their fluorescence properties.

**Figure 1:**
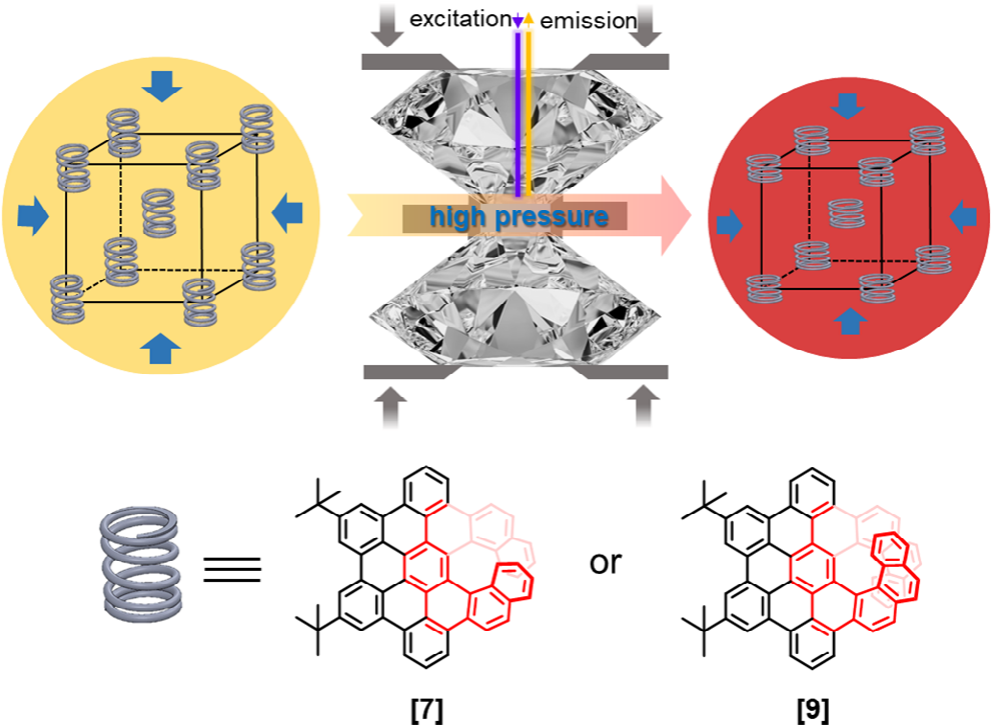
Illustration of applying high pressures on molecular springs **[7]** and **[9]** using a diamond anvil cell. The samples were excited by a 405 nm laser, and the emission wavelengths were detected at increasing pressures. The compression of crystal dimensions and the molecular springs lead to the color change of the crystals.

Adopting the calculation approach of Nakakuki et al.,^[^ ^3^ ^]^ the spring‐like mechanical characteristics of **[7]** and **[9]** upon compression were first investigated by simulations of their potential energy in a vacuum. The intramolecular helix distance (*r*) between the two centroids of the overlapping benzene rings at the helices was optimized to be 3.90 and 3.54 Å for **[7]** and **[9]**, respectively, (Figure S1) in the original state.^[^ ^28^ ^]^ The potential energy (*E*) was calculated to change in an approximately linear correlation to the square of distance change (*Δr*)^2^, which matched with the function of spring energy on the macroscopic level (Figure S2):

(1) $$E=\frac{1}{2}k{\left(\Delta r\right)}^{2}$$

Therefore, the force constants (*k*) of the molecular springs were determined as 27.5 and 58.1 N m^−1^ for **[7]** and **[9]**, respectively, suggesting that **[9]** was a more rigid molecular spring.

After growing the crystals of **[7]** and **[9]** by solvent diffusion,^[^ ^28^ ^]^ the crystalline samples were loaded into the chamber of the DAC device. Since **[7]** and **[9]** are emissive in the crystalline state at ambient pressure with peaks centered at 545 and 576 nm, respectively, their fluorescence spectra could be employed to monitor the compression–decompression behavior. A blue‐violet laser (405 nm) was used to excite the helicene molecules, and the hydrostatic pressure was calibrated by the ruby fluorescence spectra.^[^ ^29^ ^]^ As demonstrated in Figure 2a,d, the emission maxima of both molecules were gradually redshifted when increasing the pressure up to ∼6 GPa, accompanied by a significant decrease in the fluorescence intensity. The fluorescence spectra resumed their original state when releasing the hydrostatic pressure to ambient conditions (Figure 2b,e). The reversibility of the fluorescence response (Figure S3) indicated that the spectroscopic changes were caused by physical distortions of the molecular conformation and crystal packing rather than by chemical transformation or degradation. Besides, the same shape of the emission spectra suggested the absence of new emitting species, e.g., excimers or exciplexes. The pressure‐dependent piezochromic process could also be visually observed with the fluorescence photographs captured by a digital camera. As shown in Figure 2c,f, the crystals exhibited a gradual redshift in color and a significant darkening of brightness upon compression. This alternation was also fully recovered by releasing the pressure, consistent with the spectroscopic observations.

**Figure 2:**
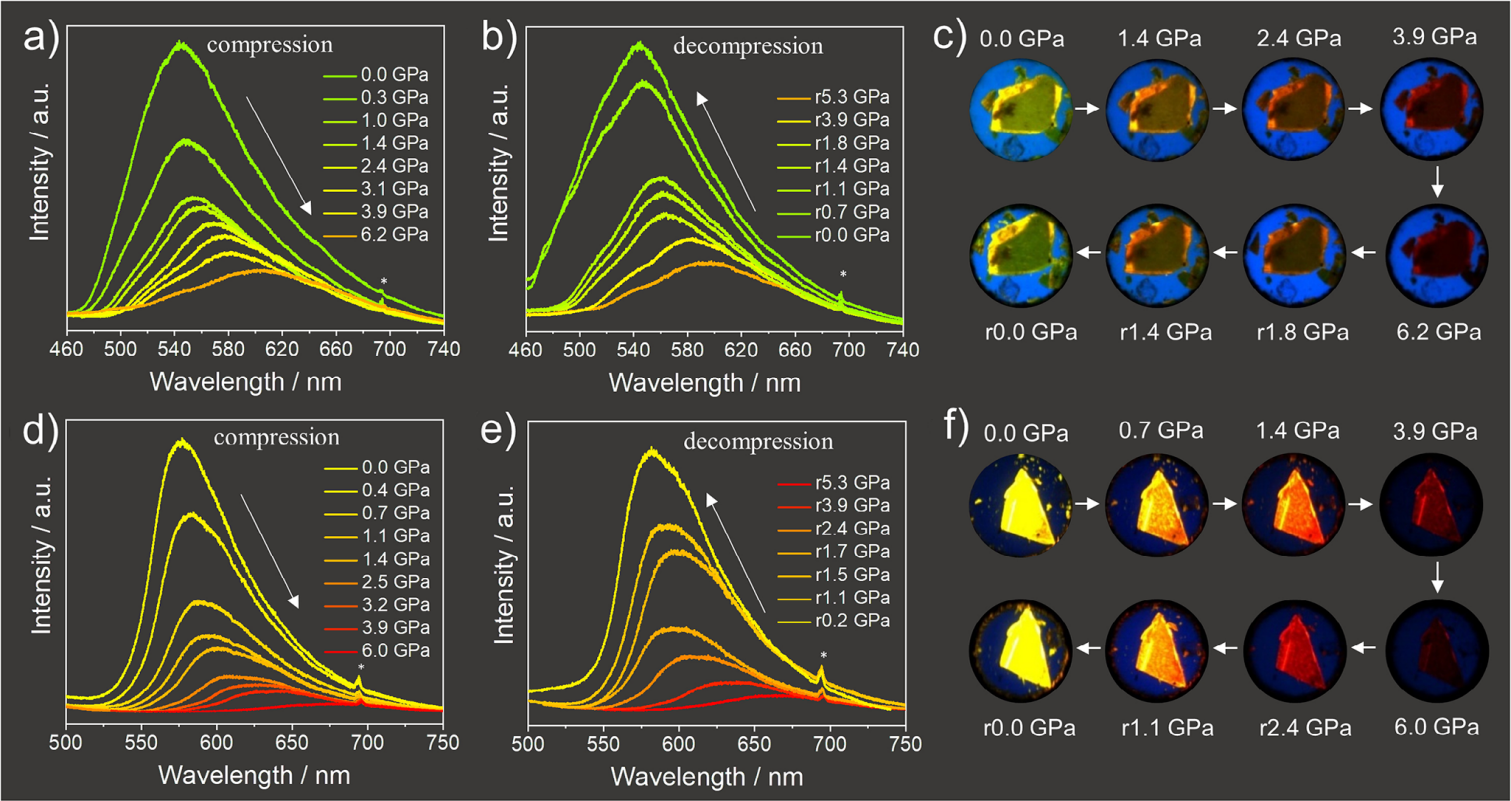
Change of fluorescence spectra of **[7]** upon a) compression and b) decompression, and c) the corresponding fluorescence images. The similar effects of **[9]** are shown in d) compression, e) decompression, and f) images. The ruby signal is marked with a star in the spectra.

More information about the pressure‐induced changes of the **[7]** and **[9]** crystals was obtained by plotting the wavelength shift and emission intensity against the hydrostatic pressures in Figures 3 and S4 based on repeated experiments. The shift of the wavelength exhibited an approximately linear relationship with the applied pressures for both molecules. The pressure‐dependent wavelength shift in **[9]** was ∼16.4 nm GPa^−1^ (−424 cm^−1^ GPa^−1^), which was larger than that of **[7]** with ∼ 10.5 nm GPa^−1^ (−309 cm^−1^ GPa^−1^). On the other hand, the fluorescence intensities decayed exponentially upon compression. The decrease in intensity was attributed to stronger intermolecular interactions at a shortened distance under higher pressures, leading to suppression of the emission process^[^ ^30^ ^]^ or additional nonradiative decay pathways.^[^ ^31^ ^]^ Notably, **[9]** possessed a larger change in both wavelength shift and intensity decrease than **[7]**. Compared with other organic piezochromic luminescent molecules in the literature,^[^ ^32^, ^33^, ^34^, ^35^, ^36^, ^37^ ^]^ these π‐extended helicenes exhibit large pressure‐dependent wavelength shifts (Figure S5), implying potential application of helicene molecules in pressure sensing devices.^[^ ^38^ ^]^

**Figure 3:**
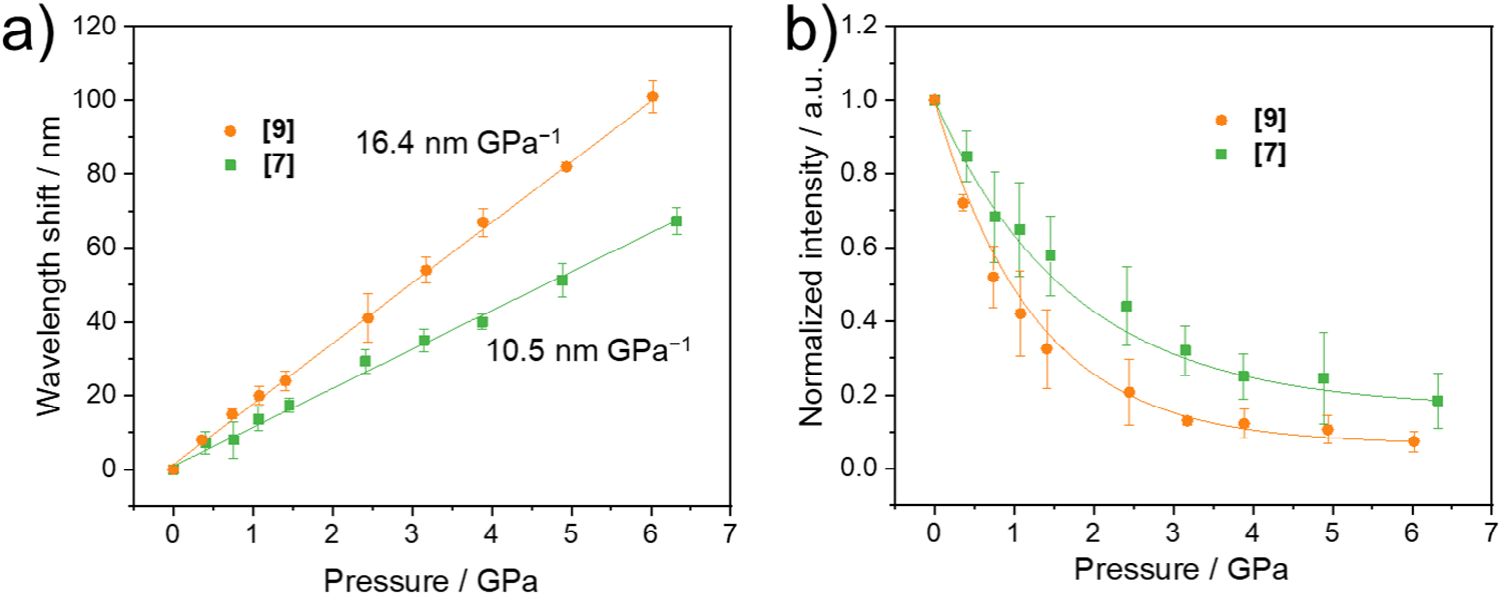
a) The shift of the fluorescence band and b) intensity change of **[7]** and **[9]** crystals under external pressures. The intensities of the peaks are normalized by the initial data from the starting pressure in the DAC. Each data point represents the mean of 3–4 independent experiments. The error bars are defined by the standard deviation.

It is widely accepted that intermolecular packing in the crystal lattices becomes closer with increasing pressures.^[^ ^14^, ^15^ ^]^ Notably, the single crystal of **[7]** contained chloroform solvent molecules, whereas **[9]** did not. The presence of small molecules in **[7]** likely influenced its intermolecular packing, thereby affecting the pressure dependence of the emission spectrum. Based on in‐depth analyses of the crystal structures (see Supporting Information), **[7]** exhibited stronger intermolecular π–π interactions with a much larger overlap of the adjacent molecules, as well as shorter intermolecular distances than **[9]** (Figure S6 and Table S1). For a planar π system, stronger π–π interactions will lead to a larger fluorescence shift.^[^ ^22^ ^]^ Considering that the π‐extended helicenes act as molecular springs, the contraction and distortion of their intramolecular structure upon compression is also expected to impact their fluorescence properties. However, it is challenging to quantitatively disentangle the inter‐ and intramolecular effects on the photophysical properties. To address this issue, the fluorescence spectra of their freeze‐dried powders and dilute solutions (10^−5^ M) were investigated at ambient pressure (Figure S7), where the intermolecular interactions were weakened or even excluded compared with the crystalline state. Freeze‐dried products typically retain a more disordered packing than their crystals and may even be amorphous.^[^ ^39^, ^40^ ^]^ Compared to the crystalline state, the emission peaks of **[7]** and **[9]** in the freeze‐dried state were hypsochromically shifted for both samples and were blueshifted even further to 495 and 532 nm in solutions, respectively. The wavelength change was likely caused by the increased intermolecular distance and thus weakened π–π interactions due to disaggregation.^[^ ^22^, ^25^ ^]^

On the other hand, high‐pressure experiments on dilute petroleum ether solutions of **[7]** and **[9]** were performed, where the photophysical properties of solvated molecules were measured to minimize the intermolecular effect. Similar to the crystals, the fluorescence maxima were gradually redshifted, and the intensity dramatically decreased upon compression (Figure S8). Compound **[9]** again exhibited a larger band displacement than **[7]**, although the changes occurred no longer in a linear way (Figure S9). Therefore, the intramolecular structural changes in the helical conformation were believed to also be relevant to the fluorescence redshift under higher pressures. The spectroscopic results in solution upon compression corroborate the general trend in crystals, suggesting additional contributions of intramolecular effects on the spectra redshift in Figure 3a.

High‐pressure synchrotron X‐ray diffraction (XRD) experiments were performed on polycrystalline powders of **[7]** and **[9]** at beamline P02.2, PETRA III at the Deutsches Elektronen‐Synchrotron (DESY). All diffraction peaks shifted to the higher 2*θ* angle region in Figure 4a,b, revealing a reduction in d‐spacings as the crystals were compressed. The changes in relative peak intensities were attributed to preferred orientations of XRD patterns when the samples became more compacted during the compression process because they filled the entire DAC chamber without a pressure transmitting medium (PTM). However, the XRD patterns of both samples exhibited good reversibility after the pressure was released (Figure S10). Notably, no new diffraction peaks were observed throughout the compression process in either sample, indicating the absence of first‐order phase transitions. To investigate the effect of the geometry structure of helicenes at high pressure on their fluorescence shift, DFT and QM/MM methods were performed to simulate the lattice parameters, electronic structures, and optical properties under pressure.^[^ ^37^, ^41^, ^42^, ^43^, ^44^, ^45^ ^]^ The unit‐cell parameters and atom positions of **[7]** and **[9]** crystals at ambient pressure were obtained from their single‐crystal XRD data,^[^ ^28^ ^]^ while those under different pressures were optimized by monotonically decreasing the unit‐cell volume with the extra pressure up to 6.0 GPa based on the Vienna ab initio simulation package.^[^ ^46^, ^47^ ^]^ The calculated evolution of lattice parameters of both samples under pressures agreed well with the Le Bail refinement of the XRD patterns (Figures S11 and S12).

**Figure 4:**
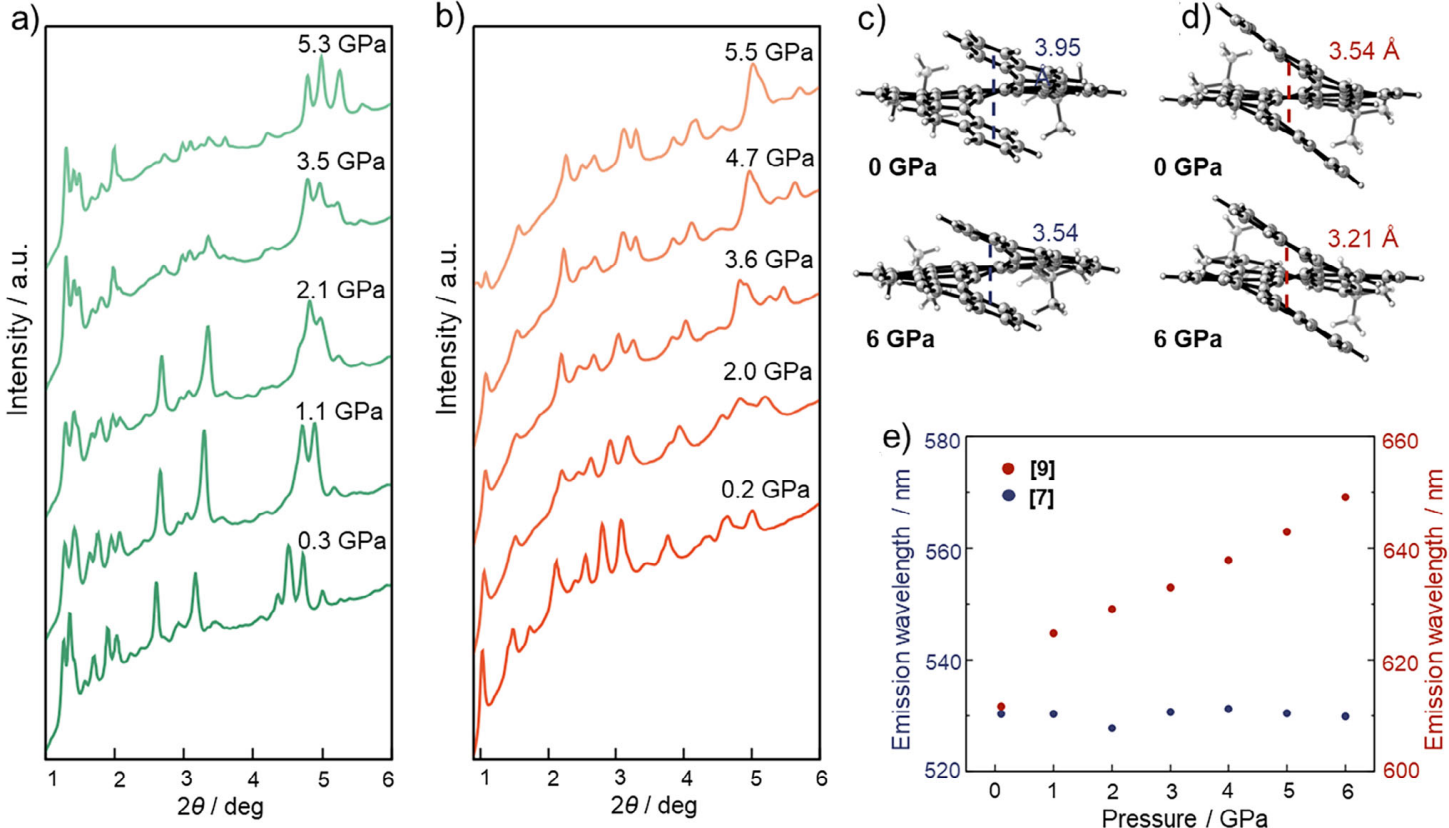
Synchrotron XRD patterns of polycrystalline powders of a) **[7]** and b) **[9]** under high pressures measured at room temperature (*λ* = 0.2903 Å). Molecular structure of c) **[7]** and d) **[9]** with denoted helix distance at ambient pressure and high pressure based on theoretical simulation. e) The theoretical emission wavelength changes of **[7]** and **[9]** as a function of pressure. Excited‐state structures were optimized at TD‐CAM‐B3LYP/6‐31G(d):UFF level of theory, while the fluorescence emission was modeled with TD‐PBE0/6‐311G(d) level of theory.

The compression of the crystal structures is expected to lead to corresponding molecular structural changes. We used the helix distance, *r*, defined as the centroid‐to‐centroid distance between overlapping benzene rings within the individual molecule to quantify these changes. At ambient pressure in the crystalline state, the *r* value for **[7]** was 3.95 Å, which was larger than that of **[9]** (3.54 Å). At 6.0 GPa, the *r* values for **[7]** and **[9]** were simulated to decrease by 0.40 and 0.33 Å, respectively (Figure 4c,d). To explore the fluorescence shift based on the contraction in these helicenes, multiscale crystal models were constructed to investigate the optoelectronic properties of **[7]** and **[9]** in the excited states using the QM/MM method in the Gaussian package^[^ ^45^, ^48^, ^49^, ^50^ ^]^ (Figure S13). By applying the pressure up to 6 GPa, the simulated fluorescence wavelength of **[9]** increased from 611 to 649 nm with a pressure‐dependent wavelength shift rate of ∼6 nm GPa^−1^ (Figure 4e). In contrast, the simulated fluorescence of **[7]** under different pressures showed no evident pressure‐dependent wavelength shift.

The changes in the fluorescence intensity were modeled by calculating the radiative rate constant *k*_r_ using the Strickler–Berg equation:^[^ ^51^, ^52^ ^]^

(2) $$k_{r}=\frac{2f{\tilde{v}}^{2}}{3}$$

where *f* was the oscillator strength and ${\tilde{v}}^{2}$ was the de‐excitation energy from S_1_ to S_0_. The calculated *k*_r_ values of **[7]** and **[9]** under different pressures were summarized in Tables S2 and S3, suggesting that the decrease in the radiative rate *k*_r_ of **[9]** upon applying pressure was larger than that of **[7]**, which explained the decreasing fluorescence intensity in Figure 3b.^[^ ^28^, ^52^ ^]^ More computational details were summarized in the Supporting Information (Figures S14–S16).

The calculation results demonstrate that the contraction and distortion of **[7]** and **[9]** helicene molecules lead to differences in the extent of their fluorescence redshifts. Electron‐hole analysis (Figure S15) shows that the excitons are located at the helix part in the excited state for both molecules, and the conjugation of the exciton becomes stronger at high pressure. These results reveal that pressure could affect electronic structure in the excited state along with the geometry structure. Therefore, the pressure sensitivity of their fluorescence properties can be attributed to the degree of π–π overlap at their helices. As depicted in Figures S1 and S14, **[9]** has a greater π–π overlap with three benzene rings at its helical ends and a shorter inter‐helix distance of 3.54 Å, compared to **[7]**, which has only one benzene ring at its helical end and a larger inter‐helix distance of 3.95 Å. Consequently, the greater and closer overlap of the benzene rings at the terminal of the helix results in a more delocalized electron distribution in the π system, making **[9]** more sensitive to the external pressure. Interestingly, as discussed above (Figure S2), **[9]** is shown to be the more rigid molecular spring because of its larger force constant. Its increased rigidity under compression may also be attributed to stronger π–π repulsion at the helix termini in **[9]**, resulting from greater overlap. However, it should be noted that the structural changes observed in molecular spring simulations may not be identical to those occurring in the crystal state. Further research is needed to elucidate the relationship between the rigidity of these molecular springs and the pressure sensitivity of their fluorescence properties.

In summary, we reported the structural changes and fluorescence behaviors of a pair of π‐extended [*n*]helicenes, **[7]** and **[9]**, as molecular springs under high pressures. In the high‐pressure fluorescence measurements, both molecules exhibited a significant redshift in emission wavelength and a decay in fluorescence intensity. Notably, **[9]** demonstrated a more sensitive fluorescence response than **[7]** in both crystalline and solvated states. Although it is not possible to fully disentangle the effects of intermolecular interaction and intramolecular structure, theoretical calculations suggest a unique effect of intramolecular structure on the emission of these spring‐like molecules. We believe that further experiments involving a wider variety of helicene molecules will establish a firm identification of their precise electronic features responsible for the different spectroscopic changes. The piezochromic behavior qualifies **[9]** for applications in sensitive pressure sensing devices due to its large emission shift rate (∼16.4 nm GPa^−1^). Our results provide new experimental insights into the structural effects of helicene molecules on their optical properties at high pressure and open up new possibilities for their use as molecular springs.

## Conflict of Interests

The authors declare no conflict of interest.

## Supporting information



Supporting Information

## Data Availability

The data that support the findings of this study are available from the corresponding author upon reasonable request.
