# Supplementary material for: Fluorescence of Helical Molecular Springs Under High Pressure
Source: Angew Chem Int Ed Engl. 2025 Mar 18;64(21):e202500923. doi: 10.1002/anie.202500923 (PMC12087869; doi:10.1002/anie.202500923)
Supplement: Supplementary file 1 — Supporting Information [file ANIE-64-e202500923-s001.pdf]

Supporting Information

Fluorescence of Helical Molecular Springs under High Pressure

Jiaxu Liang,[a] Cheng-Wei Ju,[c] Zonghang Liu,[b] Hailong Li,[a] Aigerim Karina,[f] Tobias Eklund,[a, g] Wenhao Zheng,[a] Katrin Amann-Winkel,[a, g] Weizhao Cai,[e] Manfred Wagner,[a] Zijie Qiu,[b,]* Tanja Weil,[a,]* Klaus Müllen[a,d,]*

[a] Dr. J. Liang, Dr. H. Li, Dr. W. Zheng, Prof. Dr. K. Amann-Winkel, Dr. M. Wagner, Prof. Dr. T. Weil, Prof. Dr. K. Müllen
Max Planck Institute for Polymer Research
Ackermannweg 10, 55128 Mainz, Germany
E-mail: muellen@mpip-mainz.mpg.de; weil@mpip-mainz.mpg.de

[b] Z. Liu, Prof. Z. Qiu
School of Science and Engineering, Shenzhen Institute of Aggregate Science and Technology, The Chinese University of Hong Kong, Shenzhen (CUHK-Shenzhen)
Guangdong 518172, P.R. China
E-mail: zijieqiu@cuhk.edu.cn

[c] C.-W. Ju
Pritzker School of Molecular Engineering, University of Chicago
Chicago, Illinois 60637, USA

[d] Prof. Dr. K. Müllen
Department of Chemistry, University of Cologne
Greinstr. 4-6, 50939 Cologne, Germany

[e] Prof. W. Cai
School of Materials and Energy, University of Electronic Science and Technology of China
Chengdu 611731, P.R. China

[f] A. Karina
Department of Physics, Stockholm University
Roslagstullsbacken 21, 10691 Stockholm, Sweden

[g] T. Eklund, Prof. Dr. K. Amann-Winkel
Institute of Physics, Johannes Gutenberg University Mainz
Staudingerweg 7, 55128 Mainz, Germany

Experimental Procedures

Chemicals and sample preparation

Petroleum ether was purchased from Fisher Chemical. Dimethyl sulfoxide (DMSO) was obtained from Acros Organics, methanol from VWR chemicals, and ethanol from Sigma-Aldrich Chemie GmbH.

Synthesis and crystal growth of the π-extended helicenes were conducted according to our previous report[1]. Dilute solutions (10^{-5} M) of **[7]** and **[9]** were prepared by dissolving the samples in petroleum ether. The freeze-dried samples were obtained by dissolving the molecules in DMSO before being freeze-drying.

High-pressure fluorescence measurements

The high-pressure fluorescence experiments were performed in a symmetrical DAC (Diacell® HeliosDAC from Almax easyLab). The diamond anvils were made of Type Ia diamonds with a culet size of 500 µm. Pressure was applied using a gas membrane that drove the cell pistons by being filled with argon gas. The gas pressure inside the membrane was precisely controlled by a Diacell® iGM Controller (Almax easyLab), which allowed for the application of very small incremental loads on the diamond anvils. The gas pressure A stainless steel gasket was pre-indented to around 100 µm in thickness, and then a 150 µm diameter hole was drilled with an electro discharge machine in the center as the sample chamber. A small ruby ball was placed in the chamber for *in situ* pressure calibration by monitoring the fluorescence of the ruby R1 line. The crystalline sample was then loaded in the same chamber. A mixture of methanol and ethanol (4:1) was used as the pressure transmitting medium (PTM) to generate hydrostatic pressure[2]. In the case of solution experiments, the solution was dropped into the hole of the gasket. Petroleum ether could be used as both solvent and PTM[3]. The solvents used above only gave very weak Raman signals, which were suppressed by the strong fluorescence from the samples. Both samples with and without PTM showed nearly the same spectra demonstrating the small influence of the PTM. The PTM is inert and does not react with the samples. The limit to provide hydrostatic pressure for the methanol/ethanol mixture (4:1) and petroleum ether is 10 GPa and 6 GPa, respectively[3]. Therefore, the pressures in our experiments were maintained hydrostatic and would not cause damage to the crystallinity of the samples.

Blue-violet light irradiation with the excitation wavelength of 405 nm was used as the excitation source for both ruby and the sample and the signal was collected through a 10x Mitutoyo air objective with a long working distance. The fluorescence spectra were measured using a CCD camera (PyLoN camera system from Teledyne Princeton Instruments) equipped with a spectrometer (Acton SpectraPro SP-2500). The fluorescence photographs were captured by a digital camera and a UV lamp was used for the excitation (λ_{ex} = 405 nm).

High-pressure XRD measurements

Synchrotron powder X-ray diffraction experiments were performed at beamline P02.2 of the PETRA III facility at DESY (Hamburg, Germany). We used 42.7 keV radiation (0.2903 Å). The beam was focused to around 8 (H) x 3 (V) μm^2 with a compound refractive lens system. Diffraction data were collected using a Perkin Elmer XRD1621 detector with a detector-to-sample distance of 430 mm calibrated employing a CeO_2 (SRM 674b) from NIST. The DAC was rotated up to ±5° around the axes perpendicular to the beam, and frames were collected in wide rotation mode with an acquisition time of 60s. The DAC devices and gaskets are the same as those used for the high-pressure fluorescence measurements. The opening angle of the DAC is 50º. To obtain more homogeneous XRD patterns, the samples was ground into polycrystalline powders and filled into the sample chamber without PTM. The pressure was applied by a gas membrane and measured by the ruby fluorescence method. All the experiments were conducted at room temperature. The diffraction data were analysed by the Le Bail fitting method using the GSAS-II package[4]. The fitted R values range from 0.14% to 0.33% for R_p, and from 0.28% to 0.68% for R_{wp}.

Crystal structures of [7] and [9]

The crystal structures of **[7]** and **[9]** are reported in the literature[1]. Both samples belong to the monoclinic crystal system. The space group of **[7]** is $P\ 2_1/c$ and that of **[9]** is P 2/c. The two samples possess a similar packing index with the filled space of 68.7% for **[7]** and 70.7% for **[9]**. In order to evaluate the intermolecular π-π interactions in **[7]** and **[9]**, we summarize the number and distances of all the pairs of benzene rings, which have intermolecular distances between the centroids of two rings below 4.0 Å. We find that there are 12 pairs in **[7]** and only 2 pairs in **[9]**, suggesting a much larger π-π overlap in **[7]** as shown in Figure S6. Further, **[7]** also shows shorter distances than **[9]** (Table S1). These results demonstrate that **[7]** has stronger intermolecular π-π interactions than **[9]**.

Computational methods.

Simulation of molecular springs: Atomic coordinations were obtained from the single crystal and optimized with a Gaussian16 package[5] at B3LYP[6]/6-31G(d)[7] level. Based on the optimized structure, restrictive optimization was conducted.

Ground-state structure optimization under pressure: First-principles calculations were performed using the projector augmented wave (PAW) method implemented in the Vienna ab initio simulation package (VASP)[8]. The Perdew-Burke-Ernzerhof (PBE) functional of the generalized gradient approximation (GGA) was used for electron exchange and correlation[9]. The van der Waals interactions were corrected using the DFT-D3 functional[10]. The cutoff energy for the structural relaxation was set to 500 eV and 2 × 2 × 2 k-mesh sampling was applied. The energy convergence criterion for structure relaxation was set to 10^{-5} eV. Structural relaxation was carried out until the force on each atom was less than 0.02 eV/Å. As detailed high-pressure XRD data were not available for us due to experimental limitations, the cell parameters and atom positions of **[7]** and **[9]** crystals under different pressures were optimized by monotonically decreasing the cell volume with the extra pressure up to 8.0 GPa. The continuously reduced crystal lattices with increasing pressure excluded the occurrence of sudden phase transition. Note that solvent molecules (chloroform) were presented in the crystal of **[7]** while keeping a relatively large distance away from the **[7]** molecules.

Excited-state geometry and electronic structures from QM/MM calculation: Based on the optimized crystal structure mentioned above, the electronic structures at different pressures were calculated using the Gaussian16 package[5]. The supercell (5*5*3) was extracted from the optimized crystal structures and applied in the hybrid quantum and molecular mechanics (QM/MM) model[11] to better simulate the aggregate state. The QM/MM model used in this work consisted of two layers: the central molecule was set as the high layer, calculated by high-precision at the TD-CAM-B3LYP[12]/6-31G(d) level, and the surrounding molecules were treated as a frozen low layer, computed by a UFF force field. The frozen low layer was restricted and was not optimized during the excited state structural optimization. Following the structure optimization on the excited state at the TD-CAM-B3LYP[12]/6-31G(d):UFF level, the fluorescence and electronic structure were calculated at the TD-PBE0[13]/6-311G(d)[14] level. Since our molecules were composed of hydrocarbons, the neglect of atomic charges was reasonable. In this case, the calculations of the electronic structure and energies of the excited states were only relevant to the molecular structures. This calculation method was applied in previous works[15], and the optimized structure was found to be consistent with the experimental data. With the above DFT calculation, the normal mode analysis and excited-state decay calculations were carried out. Meanwhile, GaussView, VESTA, and Chimera software were used for molecular visualization.

Excited-state decay: The non-radiative decay rates (k_{nr}) of rigid nanographene molecules with similar backbones did not show significant differences[1, 16]. Similar electronic structures, such as frontier molecular orbital (FMO) and excitation modes, could be observed in **[7]** and **[9]**

helicene monomers with different pressures. Therefore, we mainly focused on the differences in the radiative decay rate (k_r).

The lifetime τ_r (in seconds) was calculated by eq. (S1)[17]

$$\tau_r = \frac{3}{2f\tilde{v}^2} \qquad \text{(S1)}$$

where $\tilde{v}$ was the transition energy in cm^{-1}, and the oscillator strengths f were computed with the length gauge. Both two parameters were calculated with TD-DFT.

The radiative decay rate k_r was calculated by eq. (S2)

$$k_r = \frac{1}{\tau_r} \qquad \text{(S2)}$$

The radiative rate was connected with fluorescence intensity by PLQY (Φ_f) with the equation ($\Phi_f = k_r/(k_r+k_{nr})$).

In addition, the oscillator strengths were proportional to transition energy and the square of transition dipole μ as eq. (S3), which meant that the radiative rate was strongly connected with transition dipole and transition energy[16a].

$$f \propto \tilde{v}\mu^2 \qquad \text{(S3)}$$

Results and Discussion

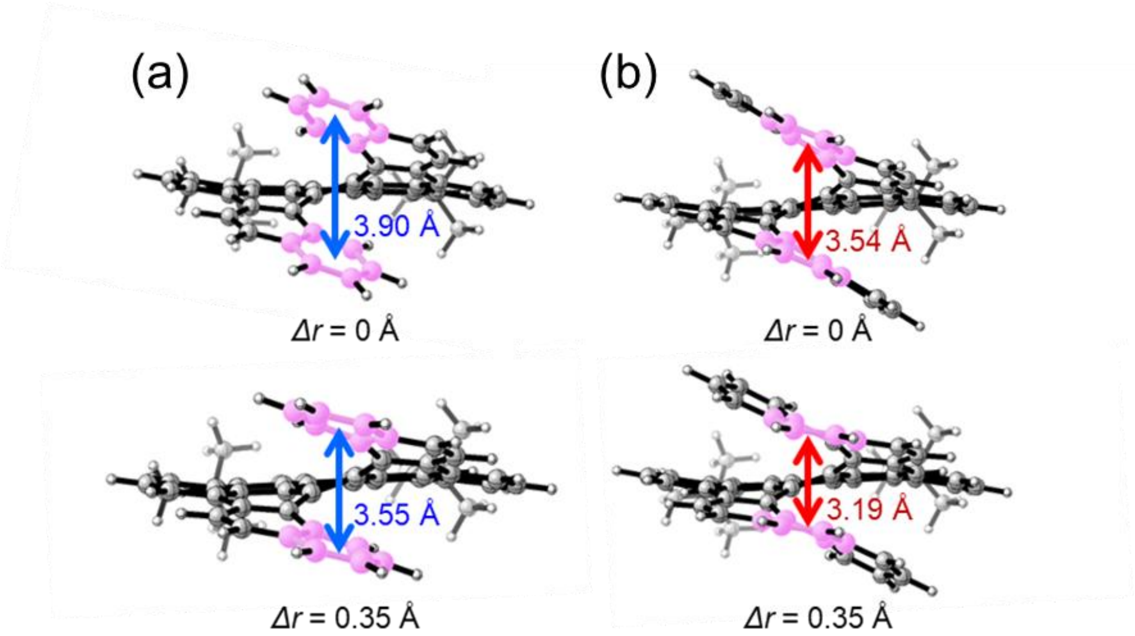

Figure S1. The distance between the two centroids of the benzene rings of (a) **[7]** and (b) **[9]** in the equilibrium state and compressed state.

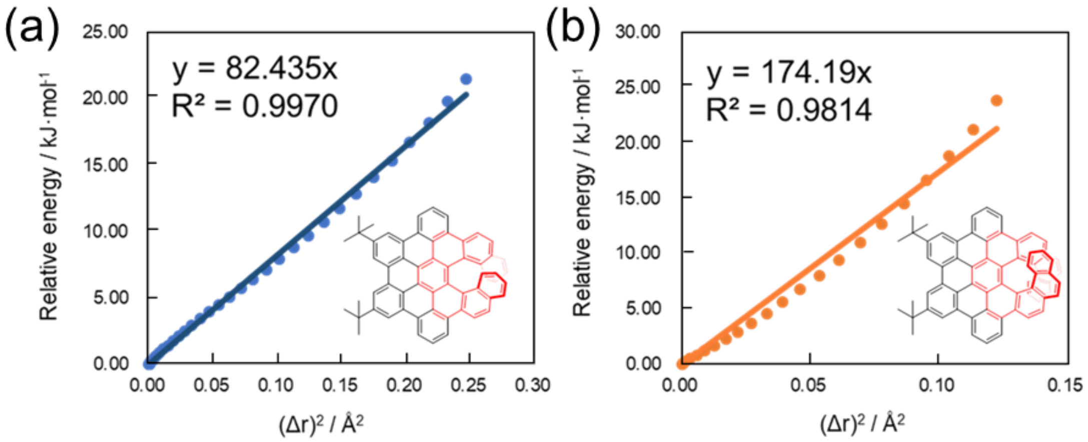

Figure S2. Increase of energy upon compression of the molecular springs of **[7]** (a) and **[9]** (b) based on DFT calculations at B3LYP/6-31G(d) level of theory with fitting results.

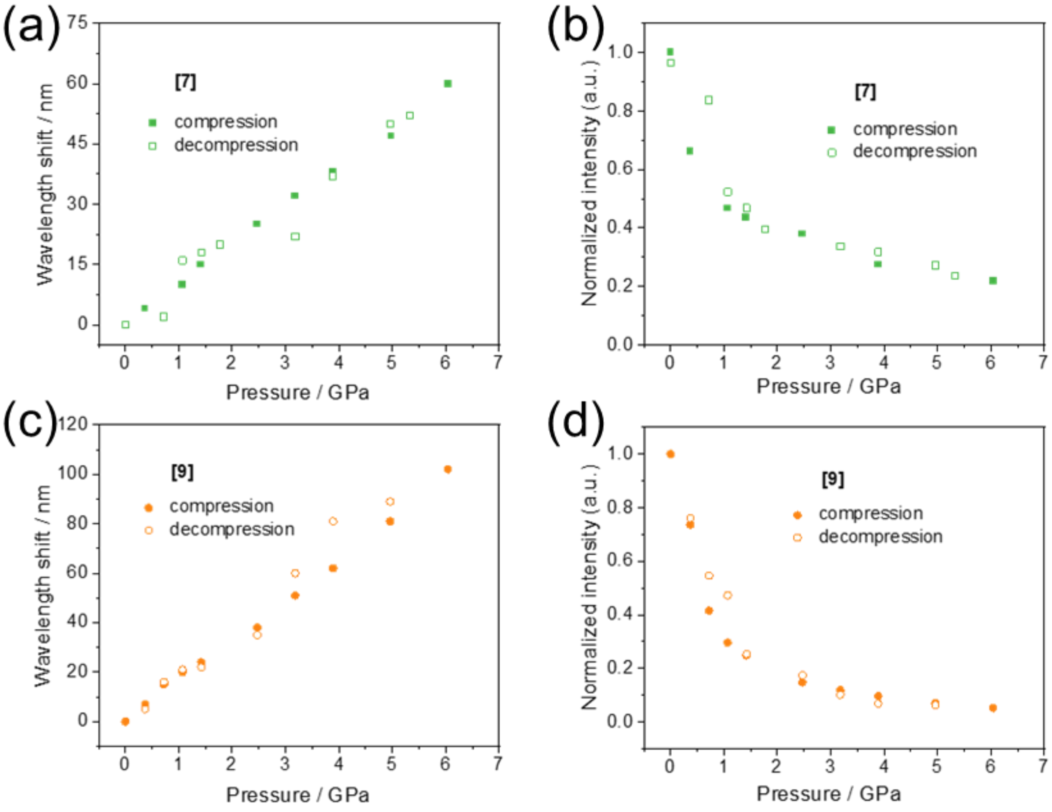

Figure S3. The fluorescence shift and intensity change of the crystals of (a,b) **[7]** and (c,d) **[9]** during the pressure cycles. The solid and empty symbols denote the data obtained during compression and decompression process, respectively.

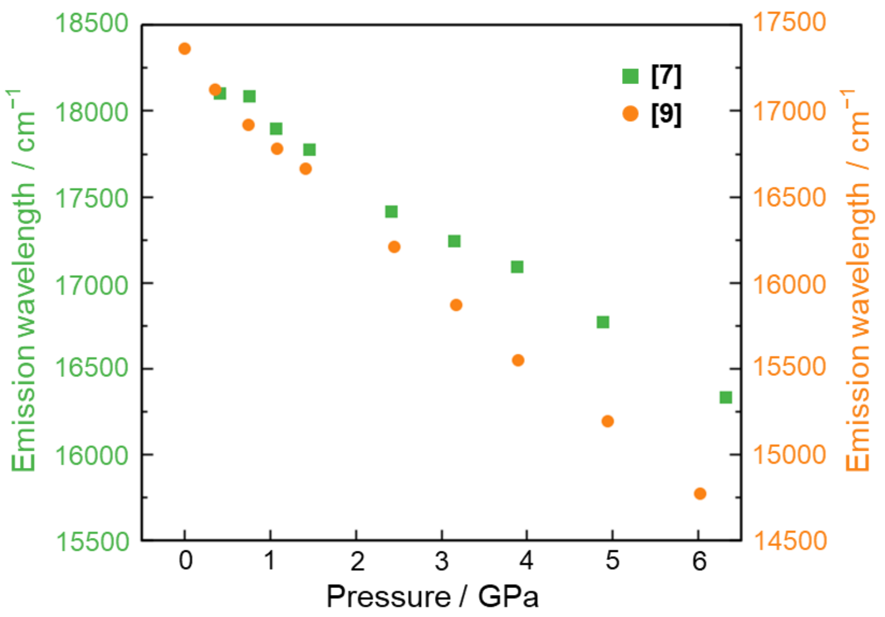

Figure S4. The wavelength shifts of **[7]** and **[9]** crystals in cm^{-1} units.

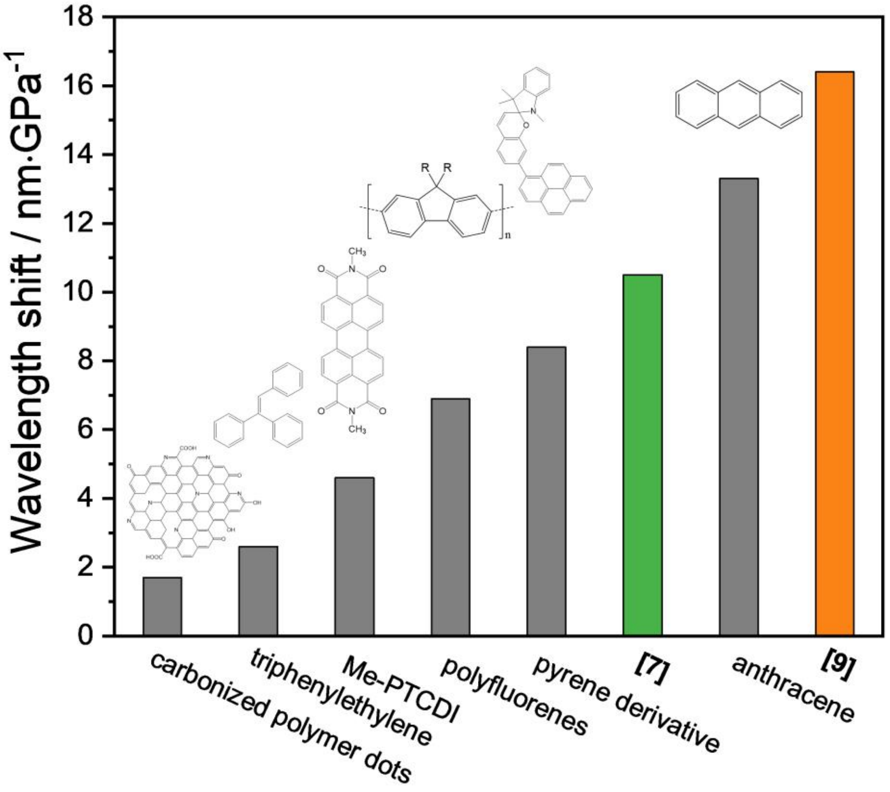

Figure S5. Comparison of slopes of fluorescence shifts of π-extended helicenes with those of aromatic molecules in the literature[18].

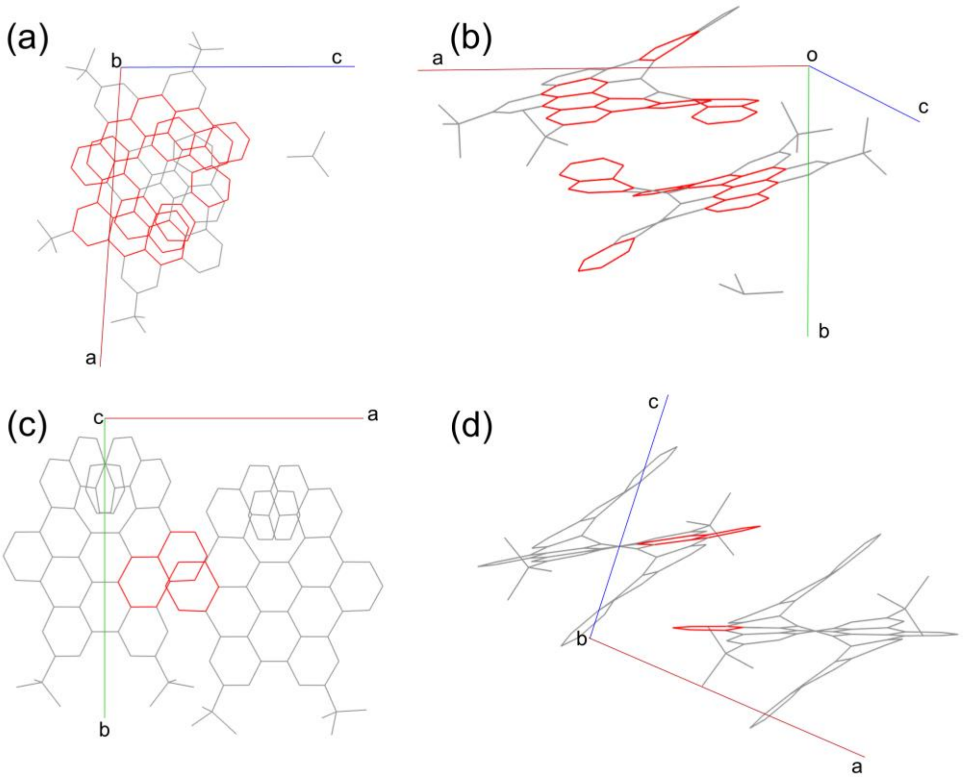

Figure S6. Single crystal structures of **[7]** (a,b) and **[9]** (c,d) viewed along different directions from XRD measurement at ambient pressure. The small molecule in **[7]** represents the solvated chloroform. The benzene ring pairs with a intermolecular distance smaller than 4.0 Å are colored in red for easy identification.

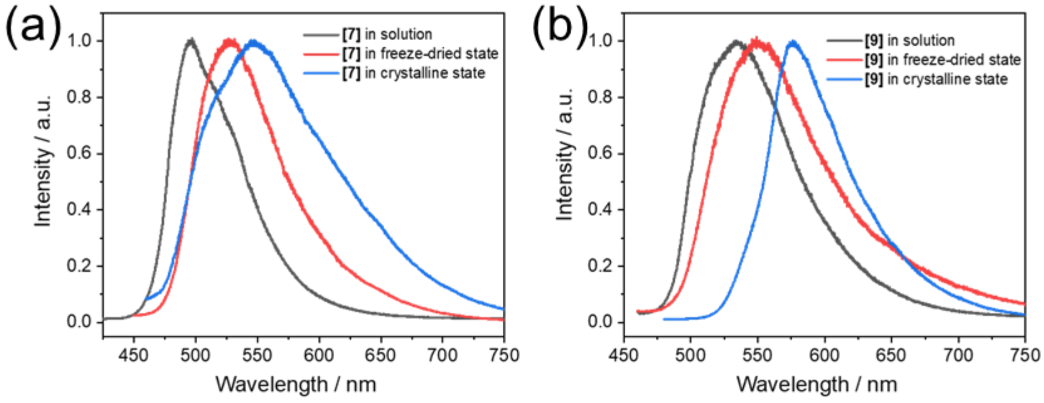

Figure S7. Fluorescence spectra of (a) **[7]** and (b) **[9]** in various states at ambient pressure.

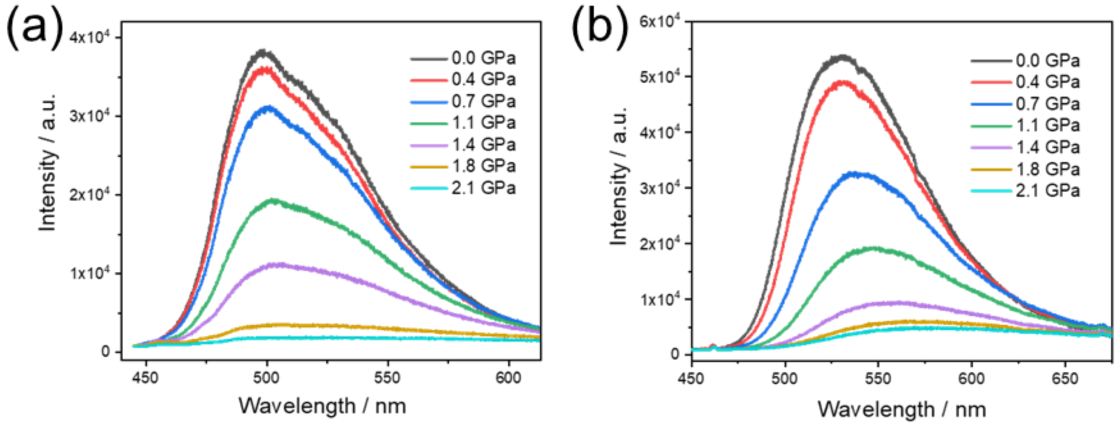

Figure S8. Fluorescence spectra change of **[7]** (a) and **[9]** (b) in petroleum ether solution upon compression.

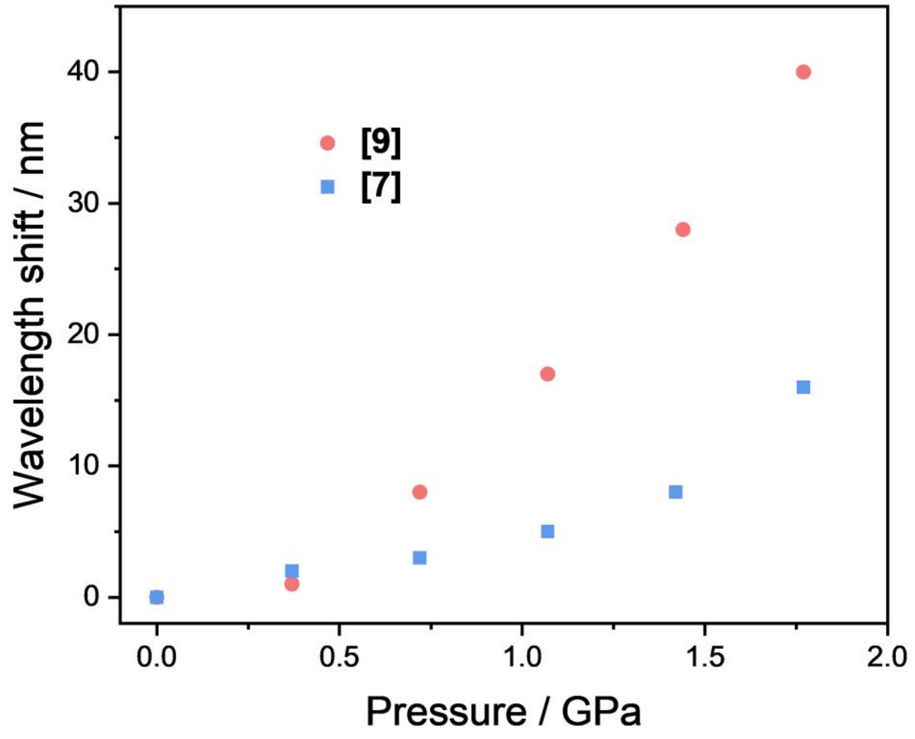

Figure S9. The fluorescence shift of **[7]** and **[9]** in petroleum ether solution under different external pressures.

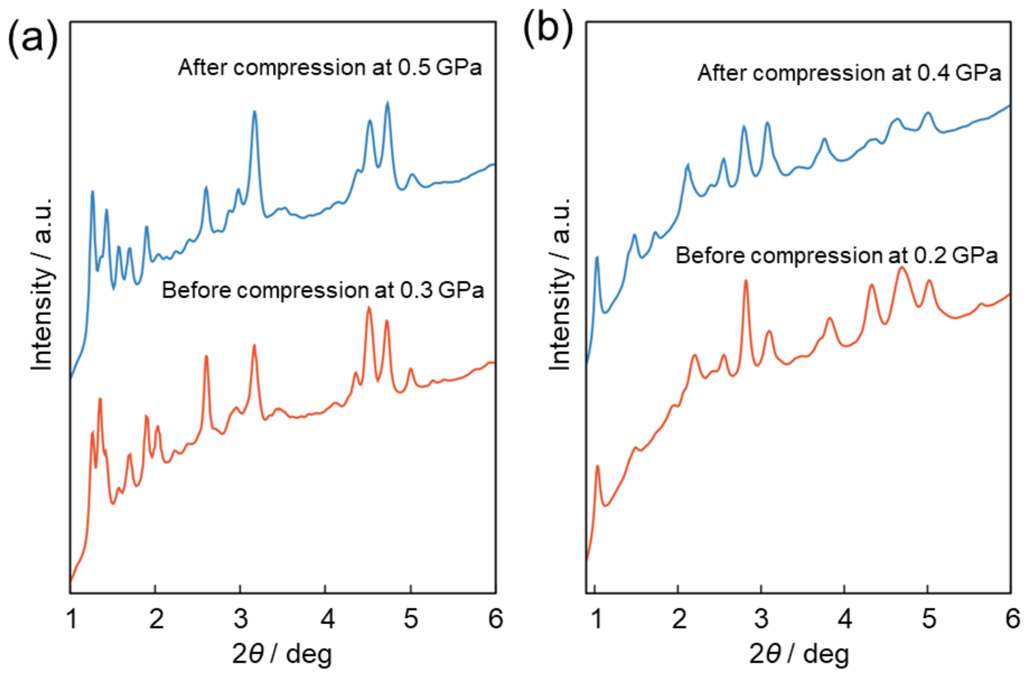

Figure S10. Synchrotron XRD patterns of polycrystalline powders of (a) **[7]** and (b) **[9]** before and after applying high pressure.

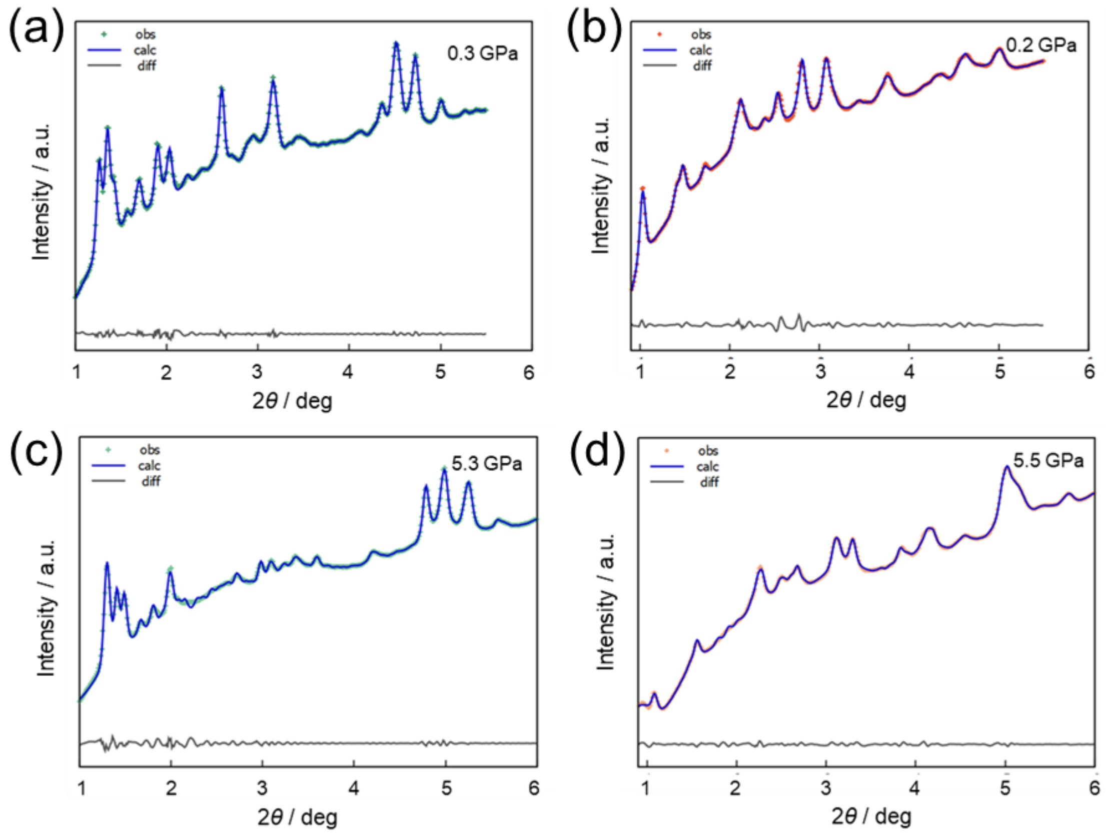

Figure S11. Le Bail fit of X-ray data of **[7]** (a, c) and **[9]** (b, d) at low and high pressure. The plus signs (obs) are the measured scattering intensity, and the blue solid line (calc) represents the fit to the data. The difference profiles are shown at the bottom with grey lines (diff).

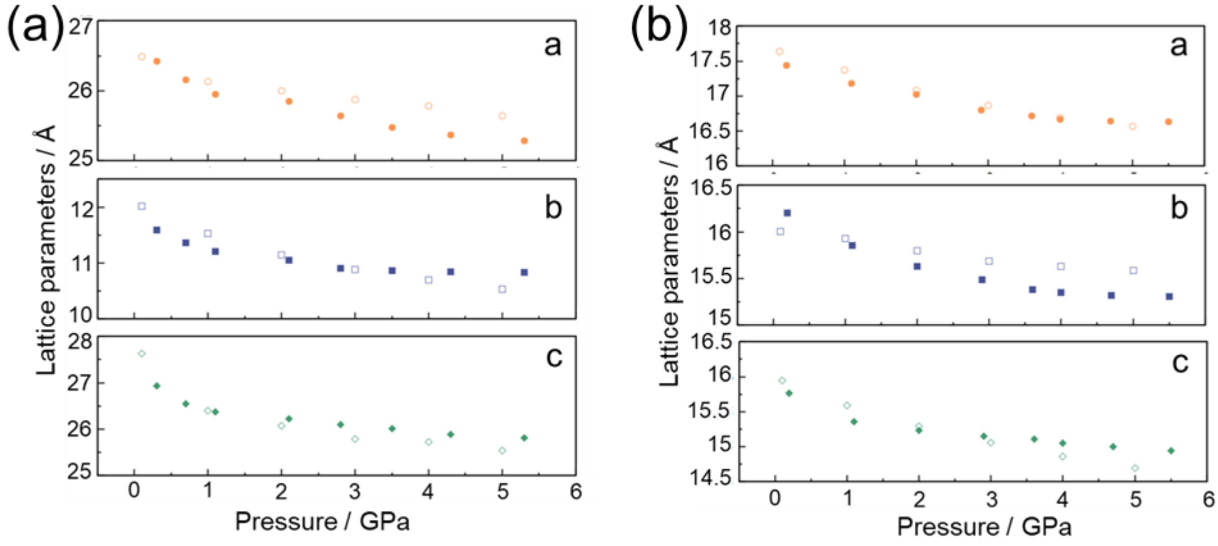

Figure S12. Evolution of lattice parameters (a,b,c lengths) of (a) **[7]** and (b) **[9]** polycrystalline powders as a function of pressure. Experimental and calculated data are indicated as solid and empty symbols, respectively.

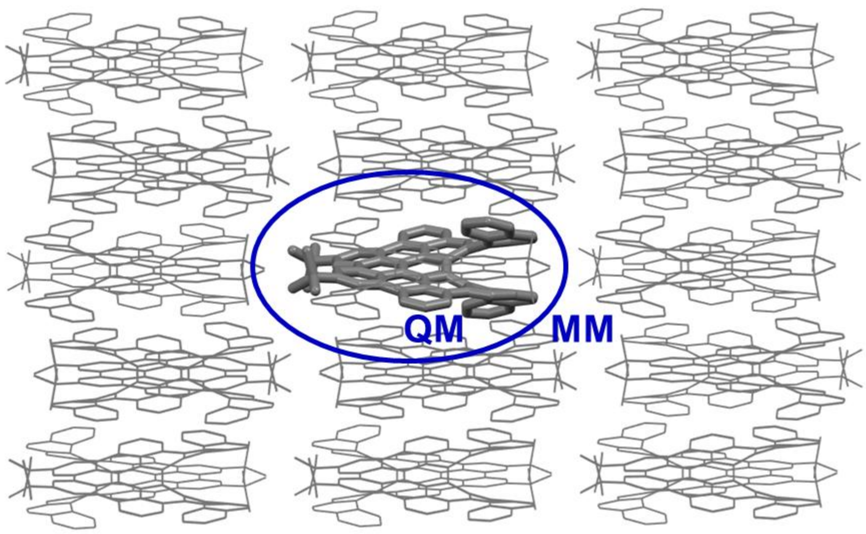

Figure S13. Illustration of the QM/MM model.

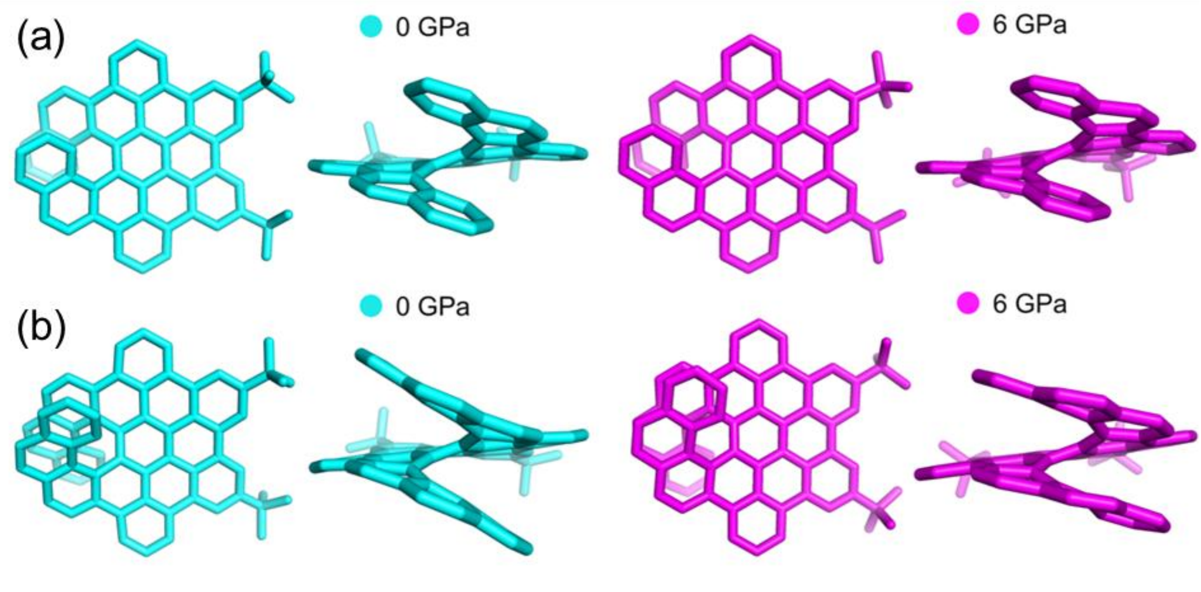

Figure S14. Excited-state structures of (a) **[7]** and (b) **[9]** under ambient pressure (0 GPa) and high pressure (6 GPa) based on TD-DFT calculation. The crystals were first optimized at different pressures with PBE-D3 using VASP, and then the molecules were optimized at the first excited state based on QM/MM method on the level of TD-CAM-B3LYP/6-31G(d):UFF.

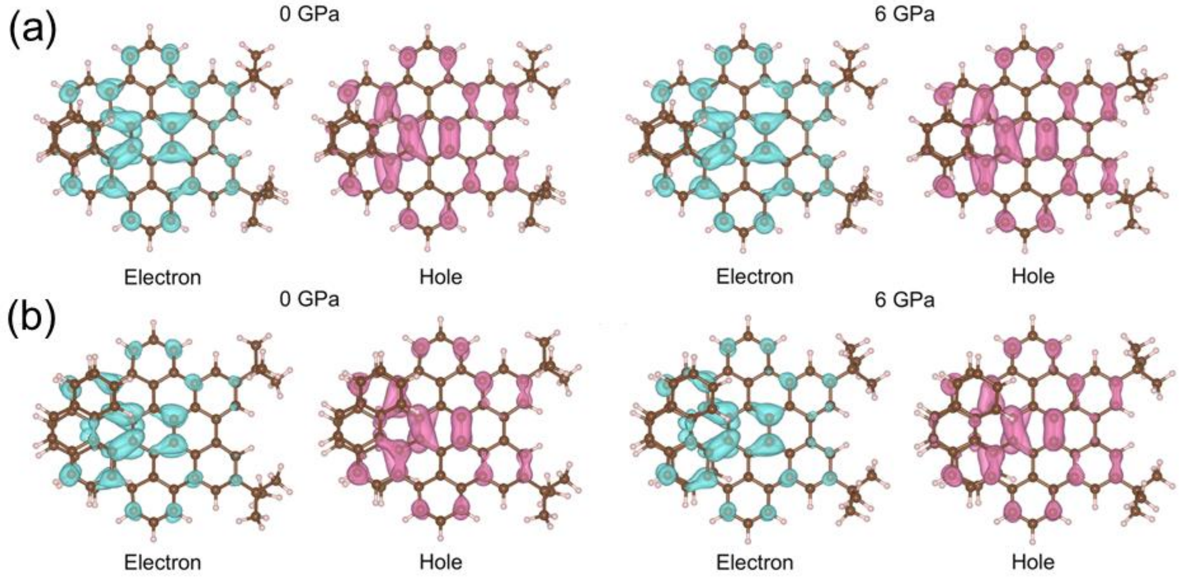

Figure S15. Electron-hole distribution of (a) **[7]** and (b) **[9]** under ambient pressure and high pressure based on TD-DFT calculations. The distributions of holes and electrons are colored in blue and purple, respectively. The crystals were first optimized at different pressures with PBE-D3 using VASP, and then the molecules were optimized at the first excited state based on QM/MM method on the level of TD-CAM-B3LYP/6-31G(d):UFF. Following the structure optimization, the electronic structure was obtained by single point calculation of optimized structure on the level of TD-PBE0/6-311G(d).

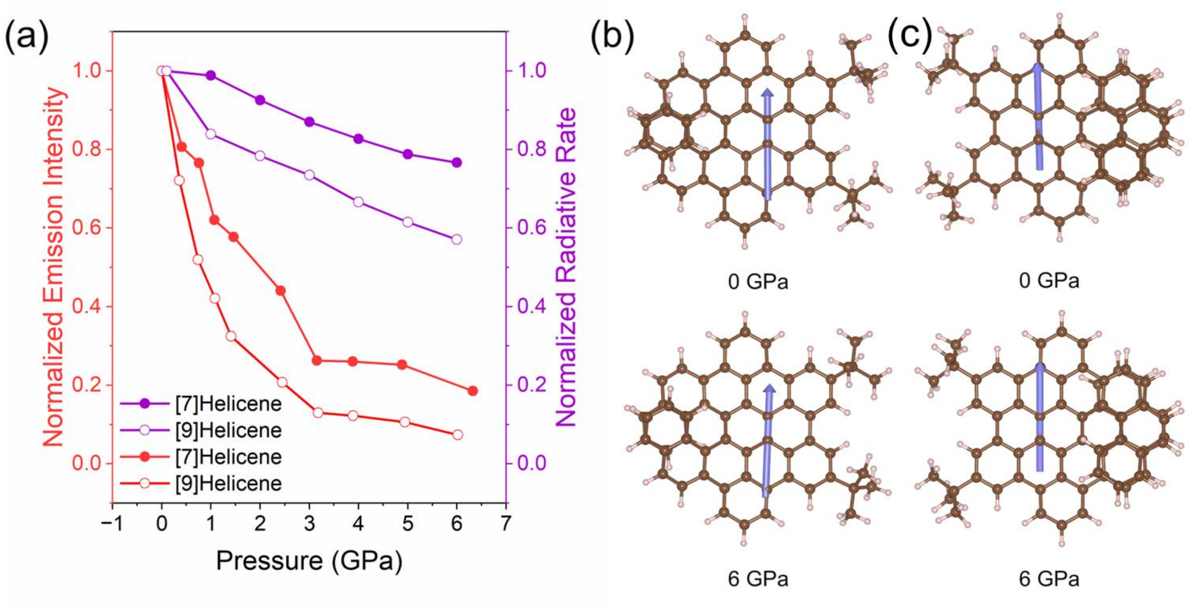

Figure S16. (a) Comparison of experimental emission intensity (red) and computational radiative rate (blue) of **[7]** and **[9]** under different pressures based on TD-DFT calculations. Transition dipole in excited state geometry of (b) **[7]** and (c) **[9]** under ambient pressure and high pressure. The crystals were first optimized at different pressures with PBE-D3 using VASP, and then the molecules were optimized at the first excited state based on the QM/MM method on the level of TD-CAM-B3LYP/6-31G(d):UFF. Following the structure optimization, the electronic structure was obtained by single point calculation of optimized structure on the TD-PBE0/6-311G(d) level.

Table S1. Analysis of intermolecular π-π interactions with Cg–Cg distances* < 4.0 Å.

[7]		[9]	
Benzene ring pair	Distance / Å	Benzene ring pair	Distance / Å
Cg(2)–Cg(33)	3.89	Cg(4)–Cg(28)	3.74
Cg(3)–Cg(31)	3.58	Cg(4)–Cg(29)	3.91
Cg(5)–Cg(33)	3.86		
Cg(5)–Cg(34)	3.81		
Cg(6)–Cg(34)	3.83		
Cg(9)–Cg(36)	3.89		
Cg(10)–Cg(27)	3.65		
Cg(10)–Cg(28)	3.56		
Cg(11)–Cg(27)	3.76		

Cg(11)–Cg(29)	3.86	
Cg(11)–Cg(30)	3.79	
Cg(13)–Cg(31)	3.89	

* Cg(X) denotes the specific benzene ring with the ring number X in the crystal unit cell from the crystal structure data which can be obtained via www.ccdc.cam.ac.uk/structures. The benzene rings involved are colored in red in Figure S6 for easy identification. The distances are defined by the distances between ring centroids. Note that intramolecular distances are excluded in the statistics.

Table S2. Calculated oscillator strength, emission energy, and radiative decay rate (k_r) of **[9]** with different pressures.

Simulated Pressure /GPa	Oscillator Strength	Emission Energy /eV	k_r /s^{-1}
0.1	0.0910	2.027	1.62×10^7
1	0.0799	1.984	1.36×10^7
2	0.0755	1.971	1.27×10^7
3	0.0715	1.956	1.19×10^7
4	0.0657	1.944	1.08×10^7
5	0.0618	1.928	9.96×10^6
6	0.0587	1.910	9.25×10^6

Table S3. Calculated oscillator strength, emission energy,and radiative decay rate (k_r) of **[7]** with different pressures.

Simulated Pressure /GPa	Oscillator Strength	Emission Energy /eV	k_r /s^{-1}
0.1	0.1749	2.338	4.15×10^7
1	0.1731	2.338	4.10×10^7
2	0.1604	2.349	3.84×10^7
3	0.1527	2.336	3.61×10^7
4	0.1451	2.334	3.43×10^7
5	0.1387	2.337	3.27×10^7
6	0.1341	2.339	3.18×10^7

References

[1] Z. Qiu, C. W. Ju, L. Frederic, Y. Hu, D. Schollmeyer, G. Pieters, K. Müllen, A. Narita, *J. Am. Chem. Soc.* **2021**, *143*, 4661 – 4667.

[2] a) G. J. Piermarini, S. Block, J. D. Barnett, *J. Appl. Phys.* **1973**, *44*, 5377 – 5382; b) R. J. Angel, M. Bujak, J. Zhao, G. D. Gatta, S. D. Jacobsen, *J. Appl. Crystallogr.* **2007**, *40*, 26 – 32.

[3] A. Katrusiak, *International Tables for Crystallography*, *Vol. H.*, *International Union of Crystallography*, **2019**, ch. 2.7, pp. 156 – 173.

[4] B. H. Toby, R. B. Von Dreele, *J. Appl. Cryst.* **2013**, 46, 544 – 549.

[5] Gaussian 16, Revision C.01, M. J. Frisch, G. W. Trucks, H. B. Schlegel, G. E. Scuseria, M. A. Robb, J. R. Cheeseman, G. Scalmani, V. Barone, G. A. Petersson, H. Nakatsuji, X. Li, M. Caricato, A. V. Marenich, J. Bloino, B. G. Janesko, R. Gomperts, B. Mennucci, H. P. Hratchian, J. V. Ortiz, A. F. Izmaylov, J. L. Sonnenberg, Williams, F. Ding, F. Lipparini, F. Egidi, J. Goings, B. Peng, A. Petrone, T. Henderson, D. Ranasinghe, V. G. Zakrzewski, J. Gao, N. Rega, G. Zheng, W. Liang, M. Hada, M. Ehara, K. Toyota, R. Fukuda, J. Hasegawa, M. Ishida, T. Nakajima, Y. Honda, O. Kitao, H. Nakai, T. Vreven, K. Throssell, J. A. Montgomery Jr., J. E. Peralta, F. Ogliaro, M. J. Bearpark, J. J. Heyd, E. N. Brothers, K. N. Kudin, V. N. Staroverov, T. A. Keith, R. Kobayashi, J. Normand, K. Raghavachari, A. P. Rendell, J. C. Burant, S. S. Iyengar, J. Tomasi, M. Cossi, J. M. Millam, M. Klene, C. Adamo, R. Cammi, J. W. Ochterski, R. L. Martin, K. Morokuma, O. Farkas, J. B. Foresman, D. J. Fox, *Gaussian, Inc., Wallingford, CT*, **2016**.

[6] a) P. J. Stephens, F. J. Devlin, C. F. Chabalowski, M. J. Frisch, *J. Phys. Chem.* **1994**, *98*, 11623 – 11627; b) A. D. Becke, *J. Chem. Phys.* **1993**, *98*, 5648 – 5652; c) C. Lee, W. Yang, R. G. Parr, *Phys. Rev. B* **1988**, *37*, 785 – 789; d) S. H. Vosko, L. Wilk, M. Nusair, *Can. J. Phys.* **1980**, *58*, 1200 – 1211.

[7] a) R. Ditchfield, W. J. Hehre, J. A. Pople, *J. Chem. Phys.* **1971**, *54*, 724 – 728; b) W. J. Hehre, R. Ditchfield, J. A. Pople, *J. Chem. Phys.* **1972**, *56*, 2257 – 2261; c) J. D. Dill, J. A. Pople, *J. Chem. Phys.* **1975**, *62*, 2921 – 2923; d) M. J. Frisch, J. A. Pople, J. S. Binkley, *J. Chem. Phys.* **1984**, *80*, 3265 – 3269.

[8] a) G. Kresse, D. Joubert, *Phys. Rev. B* **1999**, *59*, 1758 – 1775; b) G. Kresse, J. Furthmüller, *Phys. Rev. B* **1996**, *54*, 11169 – 11186.

[9] J. P. Perdew, K. Burke, M. Ernzerhof, *Phys. Rev. Lett.* **1996**, *77*, 3865 – 3868.

[10] S. Grimme, J. Antony, S. Ehrlich, H. Krieg, *J. Chem. Phys.* **2010**, *132*, 154104.

[11] a) Z. Shuai, Q. Peng, *Phys. Rep.* **2014**, *537*, 123 – 156; b) A. Warshel, M. Levitt, *J. Mol. Biol.* **1976**, *103*, 227 – 249.

[12] T. Yanai, D. P. Tew, N. C. Handy, *Chem. Phys. Lett.* **2004**, *393*, 51 – 57.

[13] C. Adamo, V. Barone, *J. Chem. Phys.* **1999**, *110*, 6158 – 6170.

[14] R. Krishnan, J. S. Binkley, R. Seeger, J. A. Pople, *J. Chem. Phys.* **1980**, *72*, 650 – 654.

[15] a) X. Lu, Y. Sun, Z. Zhang, Z. Shuai, W. Hu, *Chin. Chem. Lett.* **2021**, *32*, 1233 – 1236; b) H. Liu, Y. Gu, Y. Dai, K. Wang, S. Zhang, G. Chen, B. Zou, B. Yang, *J. Am. Chem. Soc.* **2020**, *142*, 1153 – 1158.

[16] a) A. W. Kohn, Z. Lin, T. Van Voorhis, *J. Phys. Chem. C* **2019**, *123*, 15394 – 15402; b) J. M. Fernandez-Garcia, P. J. Evans, S. Medina Rivero, I. Fernandez, D. Garcia-Fresnadillo, J. Perles, J. Casado, N. Martin, *J. Am. Chem. Soc.* **2018**, *140*, 17188 – 17196.

[17] Z. Cao, Q. Zhang, *J. Comput. Chem.* **2005**, *26*, 1214 – 1221.

[18] a) Z. A. Dreger, H. Lucas, Y. M. Gupta, *J. Phys. Chem. B* **2003**, *107*, 9268 – 9274; b) K. Paudel, H. Knoll, M. Chandrasekhar, S. Guha, *J. Phys. Chem. A* **2010**, *114*, 4680 – 4688; c) X. Meng, G. Qi, X. Li, Z. Wang, K. Wang, B. Zou, Y. Ma, *J. Mater. Chem. C*, **2016**, *4*, 7584 – 7588; d) T. Geng, T. Feng, Z. Ma, Y. Cao, Y. Chen, S. Tao, G. Xiao, S. Lu, B. Yang, B. Zou, *Nanoscale* **2019**, *11*, 5072 – 5079; e) N. Li, Y. Gu, Y. Chen, L. Zhang, Q. Zeng, T. Geng, L. Wu, L. Jiang, G. Xiao, K. Wang, B. Zou, *J. Phys. Chem. C* **2019**, *123*, 6763 – 6767; f) R. Li, M. Wang, H. Zhao, Z. Bian, X. Wang, Y. Cheng, W. Huang, *J. Phys. Chem. Lett.* **2020**, *11*, 5896 – 5901.
